# Analysis of Fat Graft Survival and Platelet-Rich Plasma Effects: The Transcriptomic Differences

**DOI:** 10.7759/cureus.34380

**Published:** 2023-01-30

**Authors:** Ecem E Yeğin, Mehmet E Yeğin, Buket Kosova, Ersin Gür, Urfat Nuriyev

**Affiliations:** 1 Bioinformatics, Ege University, Izmir, TUR; 2 Plastic, Reconstructive and Aesthetic Surgery, Ege University Faculty of Medicine, Izmir, TUR; 3 Medical Biology, Ege University, Izmir, TUR; 4 Plastic, Reconstructive and Aesthetic Surgery, Ege University, Izmir, TUR; 5 Computer Sciences, Ege University Faculty of Science, Izmir, TUR

**Keywords:** vasculogenesis, transcriptome, prp, inflammation, fat graft, bioinformatics

## Abstract

Introduction: Fat graft survival has been studied numerously but has not gone beyond hypothetical solutions. The molecular changes in survival of standard fat grafts and enhanced survival by platelet-rich plasma (PRP) are compared in this study to reveal the etiology that causes the loss of fat grafts after transplantation.

Materials and methods: A New Zealand rabbit’s inguinal fat pads were excised and divided into three groups: Sham, Control (C), and PRP. Each weighing 1 g, C and PRP fat were placed into the bilateral parascapular area of the rabbit. After 30 days, the remaining fat grafts were harvested and weighed (C = 0.7 g, PRP = 0.9 g). All three specimens were put into transcriptome analysis. Gene Ontology and Kyoto Encyclopedia of Genes and Genomes Analysis were done to compare the genetic pathways between the specimens.

Results: Transcriptome analysis showed similar differential expressions in Sham vs. PRP and Sham vs. C comparisons, indicating the dominance of the cellular immune response in both C and PRP specimens. C and PRP comparison resulted in inhibited migration and inflammation pathways in PRP.

Conclusion: Fat graft survival is more related to immune responses than any other physiological process. PRP enhances survival by attenuating cellular immune reactions.

## Introduction

Fat graft loss after transplantation is a significant concern. It varies between 30% and 70% in different studies [1-3]. Only studies with trial-and-error methods have been employed to match the reason for fat graft loss. To address the fat graft survival problem, studies that try different tumescent solution ingredients or that use various additives, cannulas, purification methods, or enrichment with stem cells have been published and declared to have varying success rates [1-3]. However, a clear etiology remains a mystery. The graft survival and replacement theories are still in debate as the significant remaining theories for this entity [4].

Bioinformatics methods made medical science more accurate in some diseases whose etiology had not been clarified. Conditions such as Brugada syndrome, a rare form of hypertrophic cardiomyopathy, or pontocerebellar hypoplasia anomaly were understood with these methods, leading to the development of studies for treatment strategies [5]. Among many of these methods, transcriptome analysis has become more popular over the past two decades. This state-of-the-art method "takes a picture" of the produced mRNA molecule profile of the interested biological material, statistically scrutinizes the complex data, and transfers the significant changes to visual instruments [6].

It was shown that platelet-rich plasma (PRP) could increase fat graft survival [7,8]. However, the mechanism of this survival increase is not focused and kept its mystery with some theoretical inferences. A study that compares standard grafts with grafts under the PRP effect may provide the clues needed to unravel this mystery.

Fat graft survival problem has not been addressed before with molecular studies. If we leave the adipose tissue microarray studies aside, a study focusing on molecular changes may find clues to the true etiology of the fat graft survival mechanism. In fact, among the many expected changes in gene expressions of grafted fat tissue, specific gene product screening may not be feasible. Therefore, this study aims to find such tips by employing transcriptome analysis as a pre-study.

## Materials and methods

This study was approved by the Local Ethics Committee for Animal Experiments on July 22, 2022 with approval number 2020-002. One male New Zealand rabbit weighing 3050 g was used for this study. Bilateral inguinal fat pads were harvested following anesthesia induction with a peritoneal injection of 10 ml/kg Ketamin-Xylazine combination. Fat tissue was minced and divided into three equal portions weighing 1 g each. The rest of the fat tissue was discarded. These portions were tagged as Sham, Control (C), and Experiment (PRP) groups. The Sham group was preserved in RNAlater™ Stabilization Solution (Thermo Fisher Scientific Co., Waltham, Massachusetts, USA) and put in a −80 °C refrigerator until the day of the transcriptome analysis experiment. The other two portions were placed under the skin of the bilateral parascapular area of the animal. The right side was defined as the PRP side, and the left side was defined as the C side. Three cc of blood were drawn from the auricular vein of the rabbit and centrifuged for three minutes at 3000 rpm. The PRP zone of the supernatant was drawn to an injector. After the fat grafts were placed, 1 cc of saline and 1 cc of PRP were injected into the C and PRP sides, respectively. After 30 days, the animal was re-anesthetized with the same combination, and the remaining grafts were harvested, weighed, and tagged per their groups. Both specimens were preserved with the similar method mentioned above. The subject was sacrificed afterward.

RNA extraction

On the day that transcriptome analysis was begun, the specimens were taken off the freezer, and all samples were sectioned into 20 µm slices with a cryostat. Five sections per group were picked and dipped into Trizol solution (Invitrogen, Stockholm, Sweden) with 60 ml of chloroform and iso-amyl-alcohol mixture. After stirring, the samples were centrifuged for 10 minutes at 10,000 rpm. The supernatant was transferred to another tube, and 2 ml of PelletPaint (Novagen, San Diego, CA, USA) was added as precipitant with 160 ml isopropanol. After five minutes of incubation, RNA was further precipitated with 10 minutes of centrifugation at 13,000 rpm. The precipitate was washed twice with 70% ethanol. After the removal of ethanol and 15 minutes of dehydration, 20 ml of RNaseZap™ (Thermo Fisher Scientific Co., Waltham, Massachusetts, USA) was added. The solution was transferred to a 1% agarose gel surface. Degradation and contamination of RNA were observed. The purity of RNA was controlled with a spectrophotometer (NanoPhotometer®, Implen, CA, USA). RNA integrity was assessed with the Bioanalyzer 2100 system using the RNA Nano 6000 Assay Kit (Agilent Technologies, CA, USA). One microgram of RNA per specimen was considered sufficient for sampling.

cDNA library preparation

The sequence library was prepared with the NEBNext® Ultra TM RNA Library Prep Kit for Illumina® (NEB, USA) under the manufacturer's directions. Using poly-T-oligo-bonded magnetic beads, mRNA was purified. In the beginning, fragmentation with divalent cations occurred at high temperatures using NEBNext First Level Strand Reaction Tampon (5X) (Ipswich, MA, USA). The first strand of the DNA was synthesized using random hexamer primers and M-MuLV reverse transcriptase (RNase-H). The other strand was synthesized using DNA polymerase-I and RNAse-H. After adenylation of the 3’ tips of the cDNA fragments, the fragments were prepared for hybridization with the NEBNext adaptor ligation, which contains a hairpin ring structure. The remaining sharp edges were transformed into blunt tips using exonuclease/polymerase activity. The library was purified with the AMPure XP system (Beckman Coulter, Beverly, ABD) to pick the cDNA fragments with ~150 to 200 bp length. Afterward, 3 μl USER enzyme (NEB, UK) was added to the adapter-bound cDNA fragments with known length and incubated at 37 °C for 15 minutes and at 95 °C for 5 minutes. PCR was done with Phusion High-Fidelity DNA Polymerase, Universal PCR Primers, and Index (X) Primers. Finally, PCR products were re-purified with the AMPure XP System, and library quality was assessed with the Agilent Bioanalyzer 2100 System.

Sequencing

Indexed samples were clustered with the cBot Cluster Generation System, which uses the HiSeq PE Cluster Kit cBot-HS (Illumina, San Diego, CA, USA), depending on manufacturer instructions. After clustering, library components were sequenced on the Illumina NovaSeq platform (Illumina, San Diego, CA, USA), and 150-bp readings were obtained in FASTQ format. Quality control with Q-score revealed high-quality data (Q20 and Q30 scores >94%). Mapping of the reads was done on the reference genome downloaded from *https://www.ncbi.nlm.nih.gov/genome/?term=Oryctolagus+cuniculus*.

After verification of the mapping data by Bowtie 2 per mapped read percentage and overall coverage and depth, the Integrative Genomics Viewer was employed to screen genes and annotations. Mapped gene counts were determined with the HTSeq v0.6.1 package in Python, depending on fragments per kilobase of exon per million mapped fragments (FPKM) values. After normalization of the read counts, differential expression analysis was done with the DESeq and DESeq2 packages in RStudio (Boston, MA). Genes that demonstrate a lower p-value than 0.05 were determined to have differentiated expressions. Gene ontology (GO) enrichment analysis was done with the clusterProfiler package in RStudio. The Kyoto Encyclopedia of Genes and Genomes (KEGG) enrichment analysis was done with the KOBAS program [9]. The RStudio ggplot2 package was used to visualize the data.

## Results

**Figure 1:**
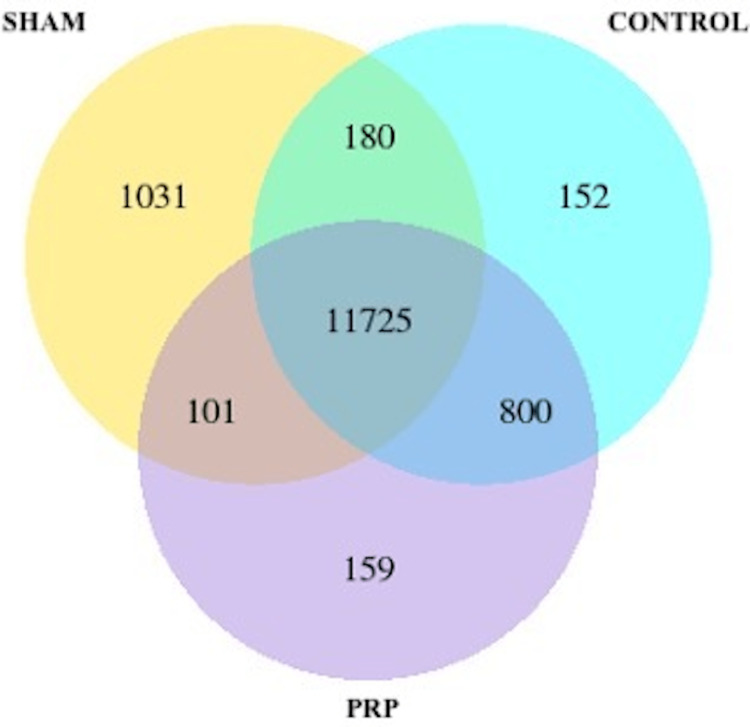
Venn diagram of gene counts in groups. These numbers refer to the total changes of expressed genes. The isolated parts refer to up or down-regulated genes. PRP: platelet-rich-plasma group.

Weight measurements revealed 0.9 and 0.7 g of remaining fat tissue in the PRP and C groups, respectively. Differentially expressed gene counts are shown in Figure 1. The Sham and C groups’ comparison revealed that 2797 genes were significantly down-regulated, while 2420 genes were significantly up-regulated (p<0.05). The PRP and C groups revealed that 2838 genes were significantly down-regulated, while 2439 genes were significantly up-regulated in the PRP group (p<0.05). The Sham and PRP groups’ comparison revealed that 2838 genes were significantly down-regulated, while 2439 genes were significantly up-regulated (p<0.05).

**Figure 2:**
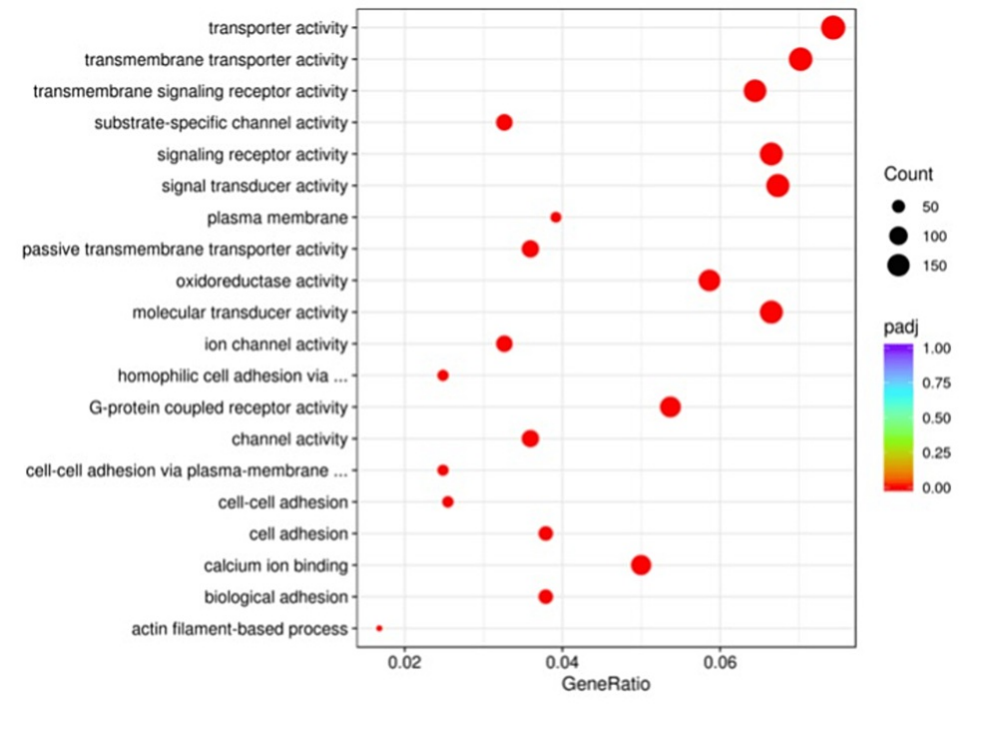
The GO analysis of differential expression between Sham and C groups. The most significant changes belong to cell membrane communication activities.

**Figure 3:**
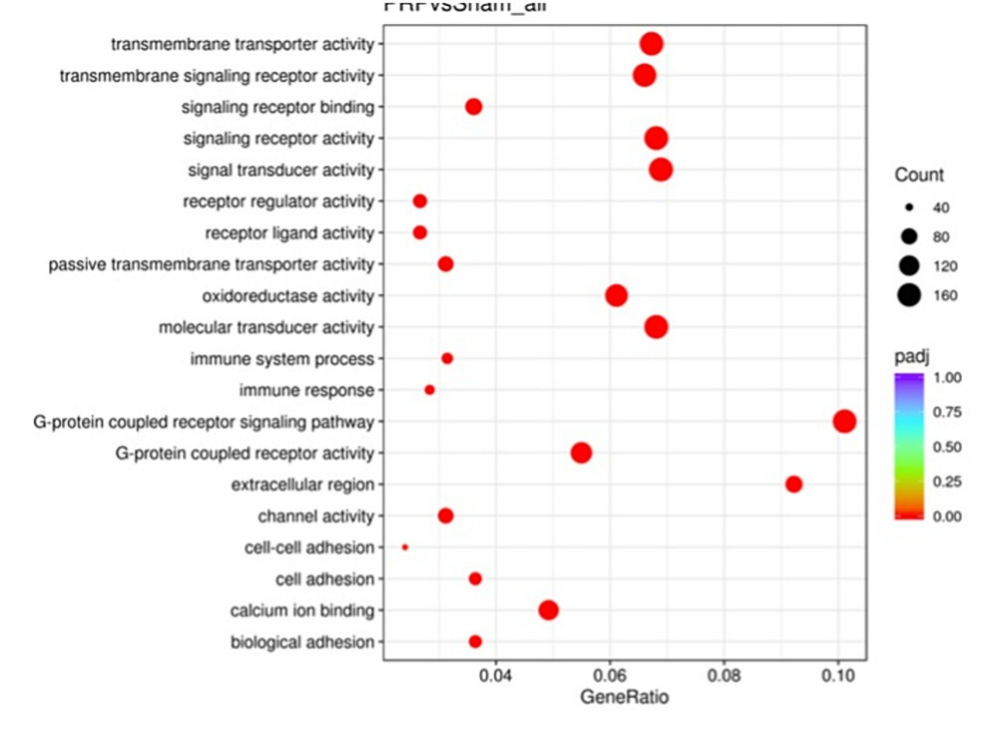
The GO analysis of differential expression between Sham and PRP groups. Most changes belong to signaling pathways, referring to cellular communications as Sama and C groups’ comparison.

**Figure 4:**
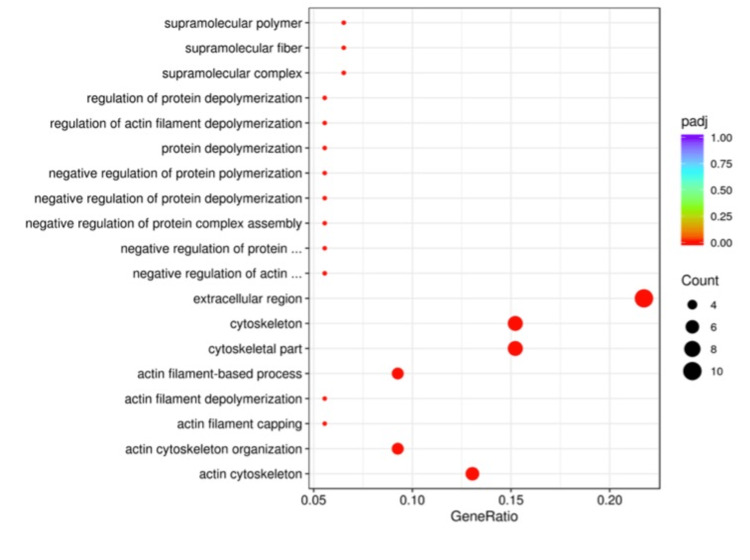
The GO analysis of differential expression between PRP and C groups. The pathway profile is changed substantially to other comparisons, including mainly molecular pathways of the cytoskeleton components.

Gene ontology analysis comparison among the differentially expressed genes between the Sham and C groups revealed significant differences in genes that are involved in transporter or receptor activity signals, oxidoreductase activity, or cell adhesion pathways (Figure 2). Similarly, the Sham and PRP groups were compared and found to have significant differences, including similar pathways as the Sham-C comparison (Figure 3). The PRP and C groups’ GO analysis comparison revealed significant differences in different pathways, including the extracellular region and cytoskeleton components (Figure 4).

**Figure 5:**
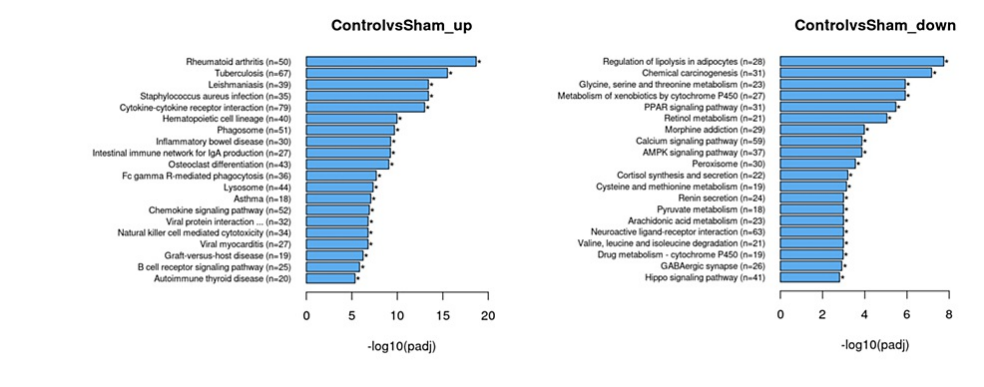
Differential expression analysis results with KEGG analysis between C and Sham groups. Note that most of the up-regulated genes of the C group are focused on type 4 hypersensitivity reactions. Down-regulated genes suggest a suppressed adipose metabolism environment. The PRP and Sham comparison was similar to these pathways, suggesting a similar state of PRP and C groups.

**Figure 6:**
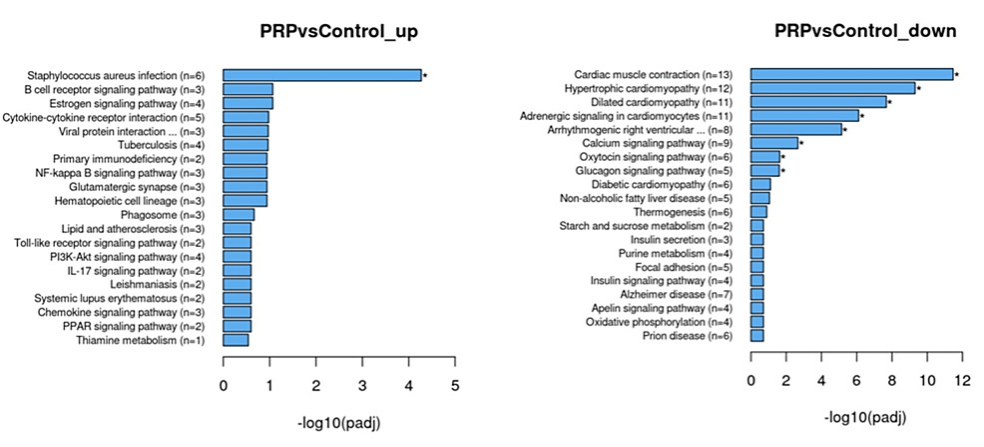
Differential expression analysis results with KEGG analysis between PRP and C groups. The lowered count of genes means that these two specimens are in similar states. Up-regulated genes are still focused on type 4 hypersensitivity reactions, but these changes sign a decreased immune response as MHC-I genes are up-regulated. Muscular pathways dominate down-regulated genes, referring to attenuated migration of cells.

Differential gene comparisons’ KEGG analyses revealed gene expression difference profiles similar to the GO analyses. The Sham and C groups' transcriptome comparison revealed that inflammatory pathways, including rheumatoid arthritis, tuberculosis, inflammatory bowel disease, etc., formed the dominant up-regulated pool. Down-regulated genes’ KEGG analysis revealed that among different ones, common metabolic pathways, including lipolysis, Cyp 450 metabolism of xenobiotics, retinol metabolism, glycine, serine, and threonine metabolism, etc. (Figure 5), were found. The KEGG analyses of PRP and Sham groups revealed similar changes in gene up- and down-regulations. PRP and C groups’ comparison revealed similar profiles of up-regulated metabolic pathways, except for 14 genes that contribute to major histocompatibility class-I (MHC-I) structure (Table 1). Down-regulated metabolic pathways included ones that primarily influence muscular or cytoskeleton contractility with very low p-values (Figure 6).

**Table 1:** The up-regulated MHC-I genes when PRP and C are compared.

MHC-class I genes	Log2 fold change
ENSOCUG00000013406	5.08436552695775
ENSOCUG00000021528	4.68343331589187
ENSOCUG00000011502	4.68343331589187
ENSOCUG00000025994	3.24617868889896
ENSOCUG00000027203	0.99190170162808
ENSOCUG00000002646	0.8427975564184
ENSOCUG00000010972	0.597016015556287
ENSOCUG00000025408	0.508199487161736
ENSOCUG00000014141	0.385880364810926
ENSOCUG00000011016	0.34453870528753
ENSOCUG00000013304	0.333706956901287
novel.299	0.294802889956508
ENSOCUG00000024751	0.253392119217591
ENSOCUG00000023680	0.0824887825813302

## Discussion

This study shows micromolecular changes in fat tissue mRNA profiles and amounts between fat tissue specimens under different conditions. The weight difference could not be supported by statistical analysis, but as the literature suggests, the higher tissue amount in the PRP group refers to higher survival [8]. Native fat tissue was found to have less inflammatory but more routine metabolic pathways active than the other specimens. Grafted fat tissue with SF injection and grafted fat tissue with PRP injection comparison revealed a dominantly downregulated muscle contractility pathway profile. These results suggest that grafted fat tissue survival depends on inflammatory cell migration, adding one more point in favor of the graft survival theory.

Differentially expressed gene profiles were found to be distinctly different in the PRP and C groups than in the Sham group. This difference was expected, as various conditions cause gene expression changes. The dominant changes in this difference give clues to the significant effectors on fat graft survival [10]. However, the dominance of inflammatory processes over angiogenic processes or stem cell proliferation was not expected as hypothesized reasons for fat graft loss previously [4]. Moreover, most of these changes imply a cellular immunogenicity dominance in the survived fat graft.

One of the suggested theories on the PRP mechanism is ischemic conditioning [11]. PRP was advocated to alter the ischemic tolerance, and its effects on many tissues were interpreted on the basis of this hypothesis. However, despite many changes in myopathy-like pathways in the differential expression analysis between the PRP and C groups, significant differences failed to imply ischemic changes such as those in the mitogen-activated protein kinase (MAPK) pathway. For instance, the only change that belongs to the MAPK pathway was the DUSP6 gene, which may also indicate changes in the cytoskeletal system [12]. The lack of gene expression data referring to the pathways related to vasculogenesis contradicts the hypothesis that vasculogenesis is the main factor affecting fat graft survival. It can also be considered that angiogenesis may improve the oxygen supply in the early postoperative period. As shown in stroke models, higher oxygen saturation in the tissue can reduce some inflammatory cell activity. However, in such circumstances, it was also demonstrated that the necrosis zone would enlarge because of higher oxidative stress and a lack of neuroprotective cytokines [13]. Besides, the cytoskeleton down-regulation may imply that immune cells, including neutrophils and macrophages, may have reduced migration, which suggests a slightly impaired immune response in the PRP group. Moreover, if a neovasculogenesis dominance was encountered, it should have traces of up-regulated vascular structure genes in all analyses.

In addition to an inflammatory response in the fat graft, the down-regulated gene expression profile of the fat graft may hint at the routine fat metabolic processes such as lipolysis, amino acid metabolism, etc. that can be attributed to a slower metabolism and preparation for an inflammatory condition. However, it can also be encountered in a cell preparing for an ischemic state. Ischemia and necrosis can also trigger inflammatory and anti-inflammatory responses.

The differentially expressed gene count difference between the PRP and C groups is 255. Eighty-seven of those have shown up-regulation, including tuberculosis, rheumatoid arthritis, Staph infection, and leishmaniasis. All these processes refer to a type 4 hypersensitivity inflammatory response in which CD8+ T cells attack the structures that fail to present native antigens via MHC-I molecules. MHC-I-related genes are up-regulated in the PRP group compared to the C group. These results can be interpreted as the PRP helping the fat grafts escape the immune response by altering antigen presentation. In other words, the hypothesis that links fat graft survival with type IV hypersensitivity reactions is more likely than any other suggestion.

A previous study by Liao et al. examined the effects of PRP on adipose tissue-derived stem cells (ADSCs). They concluded that ADSC proliferation is facilitated by growth factors in PRP while inhibiting differentiation and adipogenesis. On the other hand, as they emphasized, it was an in vitro study, limiting it to comment on the actual behavior of fat grafts in detail [14]. Besides, PRP is known to facilitate wound healing by activating macrophages. It is suggested that this may cause neovasculogenesis, but an anti-inflammatory effect was also emphasized [8].

In conclusion, this study suggests that inflammatory processes significantly affect fat graft survival. As the literature suggests, PRP can increase vascular proliferation and assist survival [15]. Still, the dominant changes in the tissue are inhibition of cellular immunity in fat graft survival, as the transcriptomic evidence of this study suggests. This result supports the "Graft Survival Theory," emphasizing the inflammatory processes as the critical problem rather than the vascular enrichment of the graft [16].

The limitations of this study include the lack of statistical information on survival rates. It is arguable because this study does not aim to justify the effects of PRP but to examine possible transcriptomic changes in fat grafts. On the other hand, a cellular component analysis with flow cytometry or histological examinations would have added value to the study. Indeed, in future studies, the authors plan to scrutinize the transcriptomic changes found in this study in correlation with such methods.

## Conclusions

Fat graft survival has been addressed by many authors, with many methods, and from many aspects. However, the etiology is still a mystery because the physiological changes behind it have not been scrutinized. In this study, molecular changes are focused on a different aspect, and it was found that a robust inflammatory response may be the main reason for fat graft loss. Future studies should focus on this hypothesis, supported by specific approaches such as microarray experiments for the target genes found in this study.

## References

[1] Rasmussen BS, Sørensen CL, Kurbegovic S (2019). Cell-enriched fat grafting improves graft retention in a porcine model: a dose-response study of adipose-derived stem cells versus stromal vascular fraction. Plast Reconstr Surg.

[2] Strong AL, Cederna PS, Rubin JP, Coleman SR, Levi B (2015). The current state of fat grafting: a review of harvesting, processing, and injection techniques. Plast Reconstr Surg.

[3] Lunger A, Ismail T, Todorov A (2019). Improved adipocyte viability in autologous fat grafting with ascorbic acid-supplemented tumescent solution. Ann Plast Surg.

[4] Pu LL (2016). Mechanisms of fat graft survival. Ann Plast Surg.

[5] Horton RH, Lucassen AM (2019). Recent developments in genetic/genomic medicine. Clin Sci (Lond).

[6] Jiang Z, Zhou X, Li R (2015). Whole transcriptome analysis with sequencing: methods, challenges and potential solutions. Cell Mol Life Sci.

[7] Modarressi A (2013). Platlet rich plasma (PRP) improves fat grafting outcomes. World J Plast Surg.

[8] Pires Fraga MF, Nishio RT, Ishikawa RS, Perin LF, Helene A Jr, Malheiros CA (2010). Increased survival of free fat grafts with platelet-rich plasma in rabbits. J Plast Reconstr Aesthet Surg.

[9] Bu D, Luo H, Huo P (2021). KOBAS-i: intelligent prioritization and exploratory visualization of biological functions for gene enrichment analysis. Nucleic Acids Res.

[10] Mattlage AE, Sutter EN, Bland MD (2019). Dose of remote limb ischemic conditioning for enhancing learning in healthy young adults. Exp Brain Res.

[11] Moog P, Kirchhoff K, Bekeran S (2020). Comparative evaluation of the angiogenic potential of hypoxia preconditioned blood-derived secretomes and platelet-rich plasma: an in vitro analysis. Biomedicines.

[12] Vo AH, Swaggart KA, Woo A (2019). Dusp6 is a genetic modifier of growth through enhanced ERK activity. Hum Mol Genet.

[13] Kawabori M, Yenari MA (2015). Inflammatory responses in brain ischemia. Curr Med Chem.

[14] Liao HT, James IB, Marra KG, Rubin JP (2015). The effects of platelet-rich plasma on cell proliferation and adipogenic potential of adipose-derived stem cells. Tissue Eng Part A.

[15] Jin R, Zhang L, Zhang YG (2013). Does platelet-rich plasma enhance the survival of grafted fat? An update review. Int J Clin Exp Med.

[16] Lindegren A, Schultz I, Sinha I (2019). Autologous fat transplantation alters gene expression patterns related to inflammation and hypoxia in the irradiated human breast. Br J Surg.

